# Biological characteristics of *Trissolcus urichi* (Crawford) (Hymenoptera: Scelionidae) on *Euschistus heros* (Fabricius) and *Dichelops melacanthus* (Dallas) (Hemiptera: Pentatomidae) Eggs

**DOI:** 10.1038/s41598-020-69406-z

**Published:** 2020-07-24

**Authors:** Ana Paula de Queiroz, Adeney de Freitas Bueno, Antônio Ricardo Panizzi, Bruna Magda Favetti, Marcela Lais Mora Grande, Pamela Gislaine Gellert Luski

**Affiliations:** 1 Universidade Federal Do Paraná, Caixa Postal 19020, Curitiba, Paraná 81531-980 Brasil; 2 Empresa Brasileira de Pesquisa Agropecuária – Embrapa Soja, Caixa Postal 231, Londrina, Paraná 86001-970 Brasil; 3 Empresa Brasileira de Pesquisa Agropecuária – Embrapa Trigo, Caixa Postal 3081, Passo Fundo, Rio Grande Do Sul 90050-970 Brasil; 4 Instituto Agronômico Do Paraná (IAPAR), Rodovia Celso Garcia Cid, km 375, Londrina, Paraná 86047-902 Brasil; 5 Universidade Estadual de Londrina, Rodovia Celso Garcia Cid, PR 445, Km 380, Londrina, Paraná 86055-900 Brasil

**Keywords:** Behavioural ecology, Entomology

## Abstract

Species of the genus *Trissolcus* are effective as egg parasitoids of *Euschistus heros* and can potentially be used in a multispecies pest management approach. However, in order to successfully use those biocontrol agents in the field, previous detailed knowledge about their life history are necessary. Therefore, we evaluate some biological characteristics of *Trissolcus urichi* on *Euschistus heros* and *Dichelops melacanthus* eggs. Three independent experiments were performed: (1) *T. urichi* host preference between *E. heros* and *D. melacanthus* eggs. (2) *T. urichi* eggs-adult period (days), number of parasitized eggs in 24 h, emergence rate (%) and sex ratio of the parasitoid in *E. heros* and *D. melacanthus* eggs. (3) Morphometric characteristics of *T. urichi* grown on *E. heros* and *D. melacanthus* eggs. *Trissolcus urichi* preferred to parasitize *E. her*os eggs, exhibiting a higher number of parasitized eggs, higher rate of emergence (%) and faster development, as well as producing progeny of larger size than the parasitoids emerged from eggs of *D. melacanthus* in relation to body length, wing length and width. Thus, it can be concluded that *T. urichi* had better performance on *E. heros* eggs, although the parasitoid had also acceptable parasitism capacity and development in *D. melacanthus* eggs.

## Introduction

Stink bugs are one of the most important groups of insects that cause yield losses in soybean (*Glycine max*) production in Brazil, Argentina and Uruguay^1^. Not only have they damage soybean in South America but also in Arkansas and other states in the Mid-South of the United States^2^. Because these insects feed directly on the soybean pods they seriously affect both yield and bean quality^1,3^. Among the recorded stink bugs from soybean fields, there have been at least 54 different species belonging to the family Pentatomidae^4^. The relative economic importance of each species might vary according to each country or its region^5^. However, of the many species of stink bug, the Neotropical Brown Stink Bug, *Euschistus heros* (Fabricius) (Hemiptera: Pentatomidae) is the most frequent pest of field crops, mainly in the central region of Brazil at latitudes between 0° and 23°^6^. In addition, more recently, the Green-Belly Stink Bug, *Dichelops melacanthus* (Dallas) (Hemiptera: Pentatomidae), has become more abundant, increasing its significance to soybean and maize (*Zea mays*) production in the Neotropical region, especially in Brazil^7^. This increase is mostly a consequence of the adopted production system in which soybean is cropped during summer immediately followed by maize in autumn. The resulting continuous food supply to the insects throughout the year, known as green bridge, has favored *D. melacanthus* outbreaks^8^.

Current stink bug management strategies in the field are primarily based on the application of pesticides^9^. However, insecticide overuse has triggered problems such as increased production costs, elimination of existing natural enemies, selection of insecticide-resistant pests, and contamination of the environment^10–12^. Therefore, a more sustainable pest management approach is urgently needed. Among the most sustainable pest management tools available, augmentative biological control (ABC) stands out due to its efficacy and worldwide acceptance, being used on more than 30 million ha globally^13^.

Egg parasitoids have been widely used in ABC and can be considered the most important stink bug biocontrol agent^14,15^. Among the egg parasitoid species, *Telenomus podisi* Ashmead (Hymenoptera: Scelionidae) is the most abundant and studied species^16–18^. However, despite being less studied, *Trissolcus urichi* (Crawford) (Hymenoptera: Scelionidae) is also among the most common egg parasitoids of stink bugs found in the Neotropical region^16,19,20^. This parasitoid species has been recorded among the most important South America soybean producers such as Brazil, Argentina, Uruguay and Paraguay as well as other countries of the Neotropical region (Mexico, Trinidad, Dominican Republic, Panama, Saint Kitts, Saint Lucia, Saint Vincent Island, Antigua and Barbados, Guyana and Bolivia) parasitizing eggs not only of *E. heros* and *D. melacanthus*, evaluated in this study but also eggs of *Acrosternum aseadum* Rolston, *Antiteuchus variolosus* Westwood, *Brontocoris nigrolimbatus* (Spinola), *Dichelops furcatus* (Fabricius), *Edessa meditabunda* Fabricius, *Edessa rufomarginata* (De Geer), *Edessa* spp., *Nezara viridula* (Linneo), *Piezodorus guildinii* (Westwood), *Tibraca limbativentris* Stal and *Thyanta perditor* (Fabricius) (Hemiptera, Pentatomidae), and *Sphyrocoris obliquus* (Hemiptera, Scutelleridae)^21^.

Despite this potential parasitism on stink bugs eggs, it is important to consider that in field conditions, it is likely that foraging *T. urichi* individuals would encounter the eggs of one host species before the eggs of another due to temporal or spatial differences in the hosts’ ovipositional activities. Therefore, this work studied *T. urichi* parasitism on *E. heros* and *D. melacanthus* eggs as well as parasitoid parasitism preference among those hosts.

## Material and methods

### Laboratory rearing of *T. urichi*, *E. heros*, and *D. melacanthus*

*Trissolcus urichi* females as well as the studied hosts, *E. heros* and *D. melacanthus*, originated from insect colonies kept at Embrapa Soybean (one of the units of the Brazilian Agricultural Research Corporation), Londrina, State of Paraná, Brazil. Colonies were kept under controlled environmental conditions inside Biochemical Oxygen Demand (BOD) climate chambers (ELETROLab®, model EL 212, São Paulo, SP, Brazil) set at 80 ± 10% humidity, temperature of 25 ± 2 °C, and a 14:10 h (L:D) photoperiod according to methodologies previously described in literature^22,23^ and briefly summarized in the followings.

*Trissolcus urichi* was collected originally from soybean fields in Embrapa Soybean Experimental Farm, Londrina, Stated of Paraná, Brazil (23° 11′ 11.7" S and 51° 10′ 46.1" W). The colony has been kept in the laboratory for approximately 3 yr. It has been reared on *E. heros* eggs (aged ≤ 24 h) glued to pieces of card (5 cm × 8 cm). When parasitoid was close to emergence (1 day before), new eggs (aged ≤ 24 h) were introduced into plastic cages (8.5 cm high and 7 cm in diameter) together with the eggs already parasitized by *T. urichi* close to parasitoid emergence. Small drops of *Apis mellifera*-produced honey were placed inside these tubes to provide food for the adults when they emerged. The tubes were then closed, and after adult emergence, the eggs allowed to be parasitized for 24 h. After 24 h, the eggs recently parasitized were removed to other cages starting a new parasitoid cycle. Adults that emerge from these eggs were used for trials as well as for colony maintenance.

Stink bug species were originally collected in soybean (*E. heros*) and maize (*D. melacanthus*) fields also in Embrapa Soybean Experimental Farm, Londrina, State of Paraná, Brazil (23° 11′ 11.7" S and 51° 10′ 46.1" W). Those populations were kept in the laboratory for approximately 4 yr during which new field insects were introduced each yr to maintain colony quality. Those insects were kept in plastic screen cages (20 cm × 20 cm sides × 24 cm tall) (Plasvale Ltda., Gaspar, State of Santa Catarina, Brazil) lined with filter paper and fed ad libitum with a mixture of beans (*Phaseolus vulgaris* L.; Fabaceae), soybeans (*Glycine max* L. Merr.; Fabaceae), peanuts (*Arachis hypogaea* L.; Fabaceae), sunflower seeds (*Helianthus annuus* L.; Asteraceae) and privet fruits (*Ligustrum lucidum* Aiton; Oleaceae). A Petri dish (diameter 9 cm) with a cotton wad soaked in distilled water was added to each cage. Cages were cleaned, food replaced, and egg masses collected on a daily basis. The eggs were then used for trials or colony maintenance.

### Bioassays

Three bioassays were conducted on *E. heros* and *D. melacanthus* eggs as follows: *Trissolcus urichi* host preference between eggs of *E. heros* and *D. melacanthus* (bioassay 1); Parasitism [egg-adult period (days), number of eggs parasitized in 24 h, emergence rate (%), and sex ratio] of *E. heros* and *D. melacanthus* eggs by *T. urichi* (bioassay 2); *Trissolcus urichi* adult morphometry when reared on *E. heros* and *D. melacanthus* eggs (bioassay 3). All trials were carried out in controlled environmental conditions inside BOD chambers (ELETROLab®, model EL 212, São Paulo, SP, Brazil) set at 25 ± 2 °C, relative humidity 80 ± 10%, and 14:10 h L:D photoperiod.

### *Trissolcus urichi* host preference between eggs of *E. heros* and *D. melacanthus* (bioassay 1)

**Figure 1:**
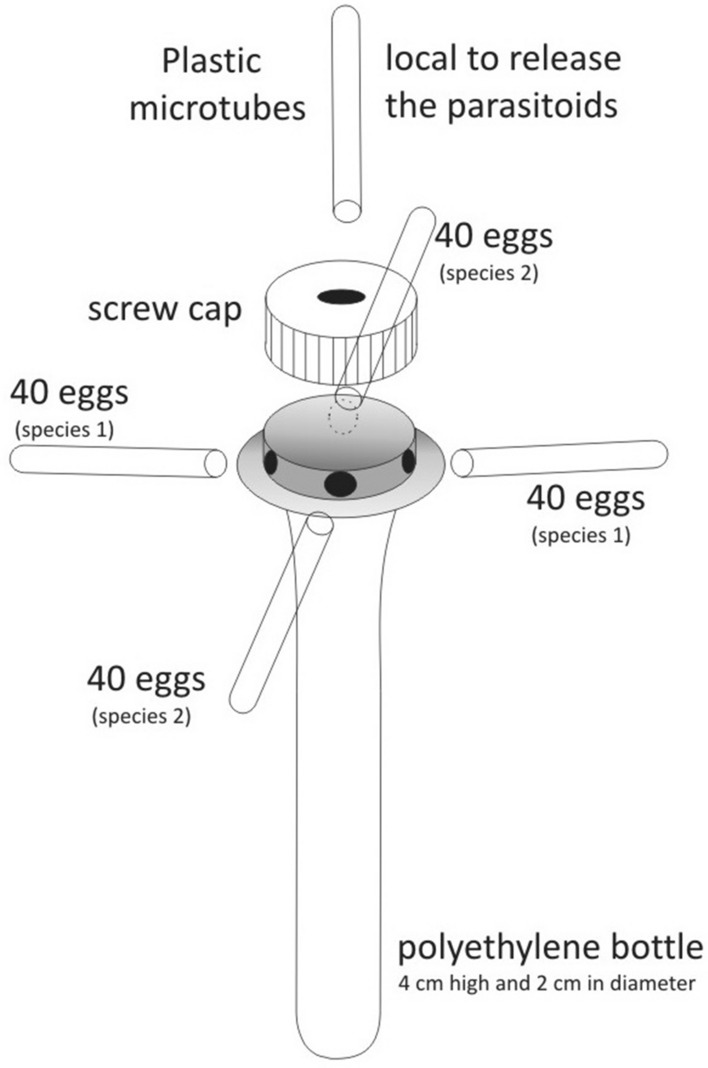
Arenas used in the host preference test of the parasitoid *Trissolcus urichi*^25^.

The host preference test was performed in a completely randomized design, with two treatments (*E. heros* and *D. melacanthus* eggs) and 15 replicates, each one using one arena with a double chance of choice (Fig. 1). The arenas were adapted from those previously described in literature^24^, composed of polyethylene bottles (4 cm high and 2 cm in diameter) and two plastic microtubes (12 mm diameter × 75 mm height) arranged equidistant at the bottom of the bottle and a microtube (12 mm diameter × 75 mm height) arranged at the top of the arena (Fig. 1)^25^.

*Euschistus heros* and *D. melacanthus* eggs; which have the average size of 0.83 mm width × 0.91 mm length for *E. heros* eggs and 0.82 mm width × 0.98 mm length for *D. melacanthus* eggs^25^; were counted (40 eggs from each host) and placed on cards (1 cm × 6 cm), and introduced into each tube on opposite sides of the arena (Fig. 1). Four mated *T. urichi* females (≤ 48 h old, mated with no previous parasitism experience) were released at the top of the arena (one female to every 40 eggs), for a period of 24 h, according to the methodology described in literature^24^ for evaluation of host preference of *Trichogramma* parasitoids and later adapted^25^ for evaluation of host preference in parasitoids of the genus *Telenomus*. A proportion of one parasitoid female was used for each 40 host eggs. After the 24-h period, cards were removed and kept in climatic chambers until emergence of adults. Preference for parasitism (%) for each host species was calculated following the equation: preference for parasitism (%) = number of eggs parasitized of each species/total number of parasitized eggs in the arena × 100. The number of parasitized eggs was calculated as the number of emerged parasitoids plus the number of adult parasitoids completely developed but dead inside the host (observed by means of dissections).

### Parasitism of* E. heros *and* D. melacanthus *eggs by* T. urichi* (bioassay 2)

The parasitism experiment was conducted in a completely randomized design with two treatments (*E. heros* and *D. melacanthus* eggs) and four replicates (each replicate composed of five females). Newly emerged *T. urichi* females (≤ 48 h old, mated with no previous parasitism experience) were individually placed in microtubes (8 cm × 2 cm) and fed with a *Apis mellifera*-produced honey droplet. Forty host eggs were glued with white glue (Tenaz®) in white card (1 cm × 6 cm) identified according to the treatments. The cards were placed in the microtubes together with the *T. urichi* females and sealed with PVC film for a period of 24 h. After this period the females were removed and the eggs kept in the same BOD chamber for later evaluation.

The biological parameters evaluated were the number of parasitized eggs, egg-adult development period (days), percentage of emergence, and sex ratio. Daily observations of progeny emergence were performed to determine the egg-adult period.

### *Trissolcus urichi* adult morphometry when reared on *E. heros* and *D. melacanthus* eggs (bioassay 3)

The experiment was conducted in a completely randomized design in a 2 × 2 factorial scheme: parasitoids from two hosts (*E. heros* and *D. melacanthus*) × two genera of the parasitoid (male and female) and 10 replicates. Ten females and 10 males of *T. urichi* progeny were analyzed for each host. For each parasitoid, morphometric measurements of the right anterior wing length and width, right posterior tibia length, and body length (head to the end of the abdomen) were performed, according the standardized quality control procedures established by the International Organization of Biological Control (Global IOBC Working Group: ‘Quality Control of Mass Reared Arthropods’)^26^. For the evaluation of these morphological characters, each specimen was photographed with a stereoscopic microscope (Leica Application Suite – Version 1.6.0) and the morphometry measured using Image J (Version 1.47)^25^.

## Data analysis

The results obtained in the experiments were submitted to exploratory analysis to evaluate the normality assumptions of the residues^27^, homogeneity of variance of treatments, and additivity of the model to allow the application of ANOVA^28^. The means were compared using the Tukey test, at 5% of error probability, using the statistical analysis program SAS^29^.

## Results

### *Trissolcus urichi* host preference between eggs of *E. heros* and *D. melacanthus* (bioassay 1)

*Trissolcus urichi* females clearly preferred *E. heros* eggs over *D. melacanthus* eggs for parasitism (F = 27.81; *p* ≤ 0.0001). The majority of parasitized eggs (63.15%) were *E. heros* eggs and only 36.85% were *D. melacanthus* eggs (Table 1).

**Table 1:** Preference of parasitism (bioassay 1) and biological characteristics (bioassay 2) of *Trissolcus urichi* on eggs of *Euschistus heros* and *Dichelops melacanthus*.

Host	Preference for parasitism (%)	Biological characteristics			
		Egg-adult period (days)^1^	Eggs parasitized (n)^1^	Emergency (%)^1^	Sex ratio^1^
*E. heros*	63.15 ± 3.53a	13.15 ± 0.05b	16.15 ± 1.09a	93.41 ± 1.38a	0.78 ± 0.02^ ns^
*D. melacanthus*	36.85 ± 3.53b	14.30 ± 0.15a	11.63 ± 1.16b	82.84 ± 3.76b	0.72 ± 0.03
CV (%)	27.32	1.35	16.19	6.43	7.4
*p*	< 0.0001	0.0004	0.0293	0.0387	0.1758
F	27.81	66.68	8.1	6.96	2.35

^1^Means ± standard error (SE) followed by the same letter within a column, did not differ significantly (Tukey test, *p* ≤ 0,05). ^ns^ANOVA non-significant.

### Parasitism of* E. heros *and* D. melacanthus *eggs by* T. urichi* (bioassay 2)

*Trissolcus urichi* egg-adult mean developmental time (days) was shorter for parasitoids reared on *E. heros* eggs (13.15 days) than for those reared on *D. melacanthus* eggs (14.30 days) (F = 66.68; *p* = 0.0004). The mean number of eggs parasitized by *T. urichi* was higher in *E. heros* (16.15 eggs or 40.38% parasitism) than in *D. melacanthus* (14.30 eggs or 35.75% parasitism) (F = 8.01; *p* = 0.0293). Similarly, parasitoid emergence (%) was higher for parasitoids reared on *E. heros* eggs (93.41%) (F = 6.96; *p* = 0.0387). The progeny sex ratio did not differ between the two hosts species (F = 2.35; *p* = 0.1758) (Table 1).

### *Trissolcus urichi* adult morphometry when reared on *E. heros* and *D. melacanthus* eggs (bioassay 3)

There was no interaction between host and sex in relation to *T. urichi* body length (F_host*sex_ = 2.43; *p*_host*sex_ = 0.1278); wing length (F_host*sex_ = 1.63; *p*_host*sex_ = 0.2097); wing width (F_host*sex_ = 2.68; *p*_host*sex_ = 0.1103) and tibia length (F_host*sex_ = 0.10; *p*_host*sex_ = 0.7519) (Table 2). *Trissolcus urichi* body length differed between the hosts (F_host_ = 34.33; *p*_host_ =  < 0.0001), with the greatest mean of body length (1.20 mm) observed for *T. urichi* that emerged from *E. heros* eggs. Likewise, differences were also observed between female and male body lengths, and female body length (1.18 mm) was greater than male body length (1.11 mm) (F_sex_ = 12.38; *p*_sex_ = 0.0012) (Table 2).

**Table 2:** Morphological characters (mm) of *Trissolcus urichi* reared on eggs of *Euschistus heros* and *Dichelops melacanthus* (bioassay 3).

Parameters	Morphological characters (mm)^1^			
	Body length	Wing length	Wing Width	Tibia length
**Host**				
*E. heros*	1.20 ± 0.02 a	1.19 ± 0.01 a	0.42 ± 0.01 a	0.34 ± 0.01^ ns^
*D. melacanthus*	1.09 ± 0.02 b	1.10 ± 0.02 b	0.39 ± 0.01 b	0.33 ± 0.01
**Sex**				
Female	1.18 ± 0.02 A	1.14 ± 0.02 A	0.41 ± 0.01 A	0.33 ± 0.01
Male	1.11 ± 0.02 B	1.14 ± 0.02 B	0.41 ± 0.01 A	0.35 ± 0.01
**Statistics**				
CV (%)	5.26	6.22	5.27	13.76
*p*_host_	< 0.0001	0.0004	0.0354	0.4853
*p*_sex_	0.0012	0.9491	0.9205	0.1667
*p*_host*sex_	0.1278	0.2097	0.1103	0.7519
F_host_	34.33	15.35	4.78	0.50
F_sex_	12.38	0.00	0.01	1.99
F_host*sex_	2.43	1.63	2.68	0.10

^1^Means ± EPM followed by the same letter in the column of each parameter (lowercase letters for host and capital letters for the genus of the parasitoid) do not differ significantly between each other (Tukey test, *p* ≤ 0,05). ^ns^ANOVA non-significant.

*Trissolcus urichi* wing length (F_host_ = 15.35; *p*_host_ = 0.0004) and wing width (F_host_ = 4.78; *p*_host_ = 0.0354) also differed between the hosts. *Trissolcus urichi* emerged from *E. heros* eggs had a greater wing length (1.19 mm) than those from *D. melacanthus* eggs (1.10 mm). The wing length (F_sex_ = 0.00; *p*_sex_ = 0.9491) and wing width (F_sex_ = 0.01; *p*_sex_ = 0.9205 respectively) did not differ between sexes. In relation to the width of the wings, there was a difference between the hosts (F_host_ = 4.78; *p*_host_ = 0.0354) with larger values for the parasitoids that emerged from eggs of *E. heros* (0.42 mm) (Table 2).

Contrary to previous reports, tibia length did not differ between hosts (F_host_ = 0.50; *p*_host_ = 0.4853), exhibiting similar length for *E. heros* (0.34 mm) and *D. melacanthus* (0.33 mm). Likewise, no differences were observed between female (0.33 mm) and male (0.35 mm) (F_sex_ = 1.99; *p*_sex_ = 0.1667) (Table 2).

## Discussion

The data presented here will contribute to understand life history of *T. urichi* and its most important biological traits regarding its parasitism on *E. heros* and *D. melacanthus* eggs. Such previous information is of theoretical and practical interest in order to later use this biocontrol agent to manage these species of stink bugs. Most of the published studies of parasitoids from the Scelionidae family report differences in their ability to parasitize depending upon the host species^15,30–32^. Overall, *T. urichi* had preference to parasitize *E. heros* eggs over *D. melacanthus* eggs. This had been previously recorded for other scelionids^19,33^, however, as far as we know this is the first report for *T. urichi*.

*Trissolcus urichi* parasitism preference for *E. heros* over *D. melacanthus* eggs might be attributed to preimaginal learning during larval development^34,35^. Thus, adults of *T. urichi* would have preferred to parasitize eggs of *E. heros* eggs because they had been previously reared on this host species. Learning might also occur in young adults^36^. Often, changes in adult behavior are induced by chemical contamination carried over from the larval to the adult environment, known as ‘chemical legacy’^37^, which occurrence can not be excluded from our trials. Furthermore, we might also speculate that *T. urichi* preference to parasitize *E. heros* eggs over *D. melacanthus* eggs observed in this study can be due to the ability of adult wasps to identify the best host, maximizing their reproductive success^38^. Different previous studies support this hypothesis, reporting that, given an abundance of hosts, parasitoids tend to avoid parasitism in hosts that present inferior nutritional qualities^39,40^. Thus, the possible better nutritional qualities of *E. heros* eggs over *D. melacanthus* eggs could be a plausible explanation to the higher performance of *T. urichi* in *E. heros* eggs, which exhibited higher number of parasitized eggs, greater emergence rate, and shorter time of egg-adult development, as well as the development of larger parasitoid adults (greater body length and greater wing length and width) when compared to results from *D. melacanthus* eggs. Even though, better nutritional value is frequently related to host size, this might not be applied to our results since *E. heros* eggs (0.83 mm width × 0.91 mm length) are smaller than *D. melacanthus* eggs (0.82 mm width × 0.98 mm length)^25^. Therefore, it is important to mention that host quality can vary not only with egg size but also with other factors such as host species^40^. Moreover, not only is nutritional quality of a host related to the physical but also chemical characteristics of each species^41,42^. Host chemical substances is known to have influence on parasitism^43^, which was not evaluated in this research. Future researches on this subject should also analyze host chemical composition of the studied hosts.

Egg-adult period (days) may indicate the quality of a specific host. The extended duration of the larva-adult period observed for *T. urichi* on *D. melacanthus* eggs reinforces the hypothesis that *D. melacanthus* eggs might be a worse nutritional host for the parasitoid. In the literature, longer larval period is described as a compensatory action to allow larvae feeding on a lower-quality host to achieve sufficient mass in order to pupate and successfully reach the adult stage^39,44^. In general, the development of insects depends on the quality of the food consumed in the juvenile stages, which may vary according to the host eggs^44^. More suitable hosts generally facilitate more rapid development of the larval phase of the parasitoid as observed for *E. heros* eggs^30^. Shorter egg-adult period can be considered a positive parasitoid feature to ABC programs, since it allows a greater number of parasitoid generations in the same time period, maximizing its control potential in the field^45^. Differences in host eggs had been previously described as an important feature for other parasitoid species (*Trichogramma* sp.)^46,47^. Different egg characteristics including surface and chorion structure, as well as changes in color during embryonic development and volume, differ between host species and may influence egg parasitism. All these peculiarities of each host species, as well as their relative differences, can affect not only *T. urichi* handling time and exploitation but also host suitability for parasitoid development, which also influences developmental time^46^.

Sex ratio is another important biological characteristic in ABC programs. The higher the proportion of females the better since they are responsible for parasitism^45^. It would had been expected that host quality would affect the sex ratio of progeny^48–50^. However, no difference in sex ratio was observed between the two hosts evaluated in this study, and both host species exhibited high proportion of females as it is desirable in ABC programs. It is important to mention that sex ratio recorded in our study was even higher than reported values from literature (0.49) for *T. urichi* parasitizing *D. melacanthus* eggs^15^. Therefore, it suggests that even though host quality differences between *E. heros* and *D. melacanthus* to *T. urichi* might exist, those differences are probably not sufficient to impact parasitoid sex ratio in *T. urichi* progeny.

Regarding the parasitoid morphometric characters evaluated in this study, it is important to mention that they are cited in the literature as good indicators of the quality of different hosts^25,51^. Therefore, the greater body length and wing length and width observed in *T. urichi* emerging from *E. heros* eggs ratifies the better nutritional conditions offered by this host than those offered by *D. melacanthus* eggs previously discussed for the comparative biology of the parasitoid in these hosts when *T. urichi* also presented greater parasitism and greater emergence of adults in *E. heros* eggs, in addition to the shorter time of egg-adult development.

When referring to the dimensions of *T. urichi* it is important to note that the female body length is greater than the male. However, there were no differences between the length and width of the wing or length of the tibia. It was observed that the size difference between males and females tends to be greater in a smaller host^52^. As only the body length of *T. urichi* varied between males and females, this may indicate that despite the apparent superiority of *E. heros* as host of *T. urichi*, this parasitoid still exhibits good development in both hosts. Overall, we can conclude that *T. urichi* had better performance (not only higher parasitism and emergence but also parasitism preference and bigger parasitoid progeny) on *E. heros* eggs compared to *D. melacanthus* eggs, although the parasitoid had also acceptable parasitism capacity and development in *D. melacanthus*. Furthermore, as mentioned earlier, this information can also be used to predict *T. urichi* dynamics when used in biological control of stink bugs in integrated pest management.

## References

[1] Panizzi AR, Schaefer CW, Panizzi AR (2000). Economic importance of stink bugs (Pentatomidae). Heteroptera of economic importance.

[2] Akin, S., Phillips, J. & Johnson, D.T. Biology, identification and management of the redbanded stink bug. Arkansas, US Cooperative Extension Service, University of Arkansas, U.S. Dept. of Agriculture, and county governments cooperating. FSA7078 (2011).

[3] Corrêa-Ferreira BS, Azevedo J (2002). Soybean seed damage by diferente species of stink bugs. Agric. For. Entomol..

[4] Panizzi AR, Slansky F (1985). Review of phytophagous pentatomids (Hemiptera: Pentatomidae) associated with soybean in the Americas. Fla. Entomol..

[5] Zerbino MS, Panizzi AR (2019). The underestimated role of pest pentatomid parasitoids in Southern South America. Arth. Plant Int..

[6] Panizzi AR, Corrêa-Ferreira BS (1997). Dynamics in the insect fauna adaptation to soybean in the tropics. Trends Entomol..

[7] Gomes EC, Hayashida R, Bueno AF (2020). *Dichelops melacanthus* and *Euschistus heros* injury on maize: Basis for re-evaluating stink bug thresholds for IPM decisions. Crop Prot..

[8] Smaniotto LF, Panizzi AR (2015). Interactions of selected species of stink bugs (Hemiptera: Heteroptera: Pentatomidae) from leguminous crops with plants in the Neotropics. Florida Entomol..

[9] Bueno AF, Bortolotto OC, Pomari-Fernandes A, França-Neto JB (2015). Assessment of a more conservative stink bug economic threshold for managing stink bugs in Brazilian soybean. Crop Prot..

[10] Sosa-Gómez, D.R., Corso, I.C. & Morales, L. Insecticide resistance to endosulfan, monocrotophos and methamidophos in the neotropical brown stink bug, *Euschistus heros* (F.) *Neotrop. Entomol.* **30**, 317–320 (2001).

[11] Sosa-Gómez DR, Silva JJD (2010). Neotropical brown stink bug (*Euschistus heros*) resistance to methamidophos in Paraná Brazil. Pesq. Agrop. Bras.

[12] Bueno AF, Batistela MJ, Bueno RCOF, José de Barros França-Neto JB, Nishikawa MAN, Libério Filho A (2011). Effects of integrated pest management, biological control and prophylactic use of insecticides on the management and sustainability of soybean. Crop Prot..

[13] van Lenteren JC, Bolckmans K, Köhl J, Ravensberg WJ, Urbaneja A (2018). Biological control using invertebrates and microorganisms: plenty of new opportunities. Biocontrol.

[14] Koppel AL, Herbert DA, Kuhar TP, Kamminga K (2009). Survey of stink bug (Hemiptera: Pentatomidae) egg parasitoids in wheat, soybean, and vegetable crops in southeast Virginia. Environ. Entomol..

[15] Laumann RA, Moraes MCB, Silva JPD, Vieira AMC, Silveira SD, Borges M (2010). Egg parasitoid wasps as natural enemies of the Neotropical stink bug *Dichelops melacanthus*. Pesq. Agropec. Bras..

[16] Corrêa-Ferreira BS, Moscardi F (1995). Seasonal occurrence and host spectrum of egg parasitoids associated with soybean stink bugs. Biol. Control..

[17] Cividanes FJ (1996). Development and emergence of *Trissolcus brochymenae* (Ashmead) and *Telenomus podisi* Ashmead (Hymenoptera: Scelionidae) at different temperatures. An. Soc. Entomol. Bras..

[18] Silva GV, Bueno AF, Neves PMOJ, Favetti BM (2018). Biological characteristics and parasitism capacity of *Telenomus podisi* (Hymenoptera: Platygastridae) on eggs of *Euschistus heros* (Hemiptera: Pentatomidae). J. Agric. Sci..

[19] Laumann RA, Moraes MCB, Pareja M, Alarção GC, Botelho AC, Maia AHN, Borges M (2008). Comparative biology and functional response of Trissolcus spp. (Hymenoptera: Scelionidae) and implications for stink bugs (Hemiptera: Pentatomidae) biological control. Biol. Control..

[20] Favetti BM, Krinski D, Butnariu AR, Loiácono MS (2013). Egg parasitoids of *Edessa meditabunda *(Fabricius) (Pentatomidae) in lettuce crop. Rev. Bras. Entomol..

[21] Margaría CB, Loiácono MS, Lanteri AA (2009). New geographic and host records for scelionid wasps (Hymenoptera: Scelionidae) parasitoids of insect pests in South America. Zootaxa.

[22] Peres WAA, Corrêa-Ferreira BS (2004). Methodology of mass multiplication of *Telenomus podisi* Ashmead and *Trissolcus basalis* (Hymenoptera: Scelionidae) on eggs of *Euschistus heros* (Hemiptera: Pentatomidae). Neotrop. Entomol..

[23] Panizzi AR, Parra JRP, Santos CH, Carvalho DR (2000). Rearing the southern green stink bug using artificial dry diet and artificial plant. Pesq. Agropec. Bras..

[24] Thuler RT, Volpe HXL, Bortoli SA, Goulart RM, Viana CLT (2007). Metodologia para avaliação da preferência hospedeira de parasitoides do gênero *Trichogramma Westood*. Bol. San. Veg..

[25] Queiroz AP, Taguti EA, Bueno AF, Grande MLM, Costa CO (2018). Host preferences of *Telenomus podisi* (Hymenoptera: Scelionidae): parasitism on eggs of *Dichelops melacanthus*, *Euschistus heros*, and *Podisus nigrispinus* (Hemiptera: Pentatomidae). Neotrop. Entomol..

[26] van Lenteren JC (2003). Quality control and production of biological control agents: theory and testing procedures 327.

[27] Shapiro SS, Wilk MB (1965). An analysis of variance test for normality (complete samples). Biometrika.

[28] Burr IW, Foster LA (1972). A Test for Equality of Variances.

[29] Institute SAS (2009). SAS User’s Guide: Statistics, Version 8e.

[30] Sujii ER, Costa MLM, Pires CSS, Colazza S, Borges M (2002). Inter and intra-guild interactions in egg parasitoid species of the soybean stink bug complex. Pesq. Agropec. Bras..

[31] Zhou Y, Abram PK, Boivin G, Brodeur J (2014). Increasing host age does not have the expected negative effects on the fitness parameters of an egg parasitoid. Entomol. Exp. Appl..

[32] Jones TS, Bilton AR, Mak L, Sait SM (2015). Host switching in a generalist parasitoid: contrasting transient and transgenerational costs associated with novel and original host species. Ecol. Evol..

[33] Orr DB (1988). Scelionid wasps as biological control agents: a review. Florida Entomol..

[34] Blackiston DJ, Casey ES, Weiss MR (2008). Retention of memory through metamorphosis: can a moth remember what it learned as a caterpillar?. PlosOne.

[35] Kaiser L, Pham-Delegue MH, Masson C (1989). Behavioural study of plasticity in host preferences of *Trichogramma maidis* (Hymenoptera: Trichogrammatidae). Physiol. Entomol..

[36] Gandolfi M, Mattiacci L, Dorn S (2003). Preimaginal learning determines adult response to chemical stimuli in a parasitic wasp. Proc. R. Soc. Lond. B.

[37] Corbet SA (1985). Insect chemosensory responses: a chemical legacy hypothesis. Ecol. Entomol..

[38] Pluke RWH, Leibee GL (2006). Host preferences of *Trichogramma pretiosum* and the influence of prior ovipositional experience on the parasitism of *Plutella xylostella* and *Pseudoplusia includes* eggs. Biocontrol.

[39] Stephens DW, Krebs JR (1986). Foraging theory.

[40] Vinson SB, Iwantsch GF (1980). Host suitability for insect parasitoids. Annu. Rev. Entomol..

[41] Bin F, Vinson SB, Strand MR, Colazza S, Jones WA (1993). Source of an egg kairomone for *Trissolcus basalis*, a parasitoid of *Nezara viridula*. Physiol. Entomol..

[42] Borges M, Costa MLM, Sujii ER, Cavalcanti MDG, Redigolo GF, Resck IS, Vilela EF (1999). Semiochemical and physical stimuli involved in host recognition by *Telenomus podisi* (Hymenoptera: Scelionidae) toward *Euschistus heros* (Heteroptera: Pentatomidae). Physiol. Entomol..

[43] Borges M, Aldrich JR (1994). Attractant pheromone for Nearctic stink bug, *Euschistus obscurus* (Heteroptera: Pentatomidae): insight in to a Neotropical relative. J. Chem. Ecol..

[44] Pomari AF, Bueno AF, Bueno RCOF, Menezes Junior AO (2012). Biological Characteristics and thermal requirements of the biological control agent *Telenomus remus* (Hymenoptera: Platygastridae) reared on eggs of different species of the genus *Spodoptera* (Lepidoptera: Noctuidae). Ann. Entomol. Soc. Am..

[45] Bueno RCO, Parra JRP, Bueno AF (2009). Biological characteristics and thermal requirements of a Brazilian strain of the parasitoid *Trichogramma pretiosum* reared on eggs of *Pseudoplusia includes* and *Anticarsia gemmatalis*. Biol. Control..

[46] Cônsoli FL, Kitajima EW, Parra JRP (1999). Ultrastructure of the natural and factitious host eggs of *Trichogramma galloi* Zucchi and *Trichogramma pretiosum* Riley (Hymenoptera: Trichogrammatidae). Int. J. Insect. Morphol. Embriol..

[47] Bai B, Luck RF, Forster L, Stephens B, Janssen JAM (1992). The effect of host size on quality attributes of the egg parasitoid *Trichogramma pretiosum*. Entomol. Exp. Appl..

[48] Schwartz A, Gerling D (1974). Adult biology of *Telenomus remus* (Hymenoptera: Scelionidae) under laboratory conditions. Entomophaga.

[49] Charnov EL, Los-Den Hartogh RL, Jones WT, Van Den Assem J (1981). Sex ratio evolution in a variable environment. Nature.

[50] Houseweart MW, Jennings DT, Welty C, Southard SG (1983). Progeny production by *Trichogramma minutum* (Hymenoptera: Trichogrammatidae) utilizing eggs for *Choristoneura fumiferana* (Lepidoptera: Tortricidae) and *Sitotroga cerealella* (Lepidoptera: Gelechiidae). Can. Entomol..

[51] Sequeira R, Mackauer M (1992). Covariance of adult size and development time in the parasitoid wasp *Aphidius ervi* in relation to the size of its host *Acyrthosiphon pisum*. Evol. Ecol..

[52] Mackauer M (1996). Sexual size dimorphism in solitary wasps: influence of host quality. Oikos.

